# Persistence, clearance and reinfection regarding six high risk human papillomavirus types in Colombian women: a follow-up study

**DOI:** 10.1186/1471-2334-14-395

**Published:** 2014-07-16

**Authors:** Sara C Soto-De León, Luisa Del Río-Ospina, Milena Camargo, Ricardo Sánchez, Darwin A Moreno-Pérez, Antonio Pérez-Prados, Manuel E Patarroyo, Manuel A Patarroyo

**Affiliations:** 1 Molecular Biology and Immunology Department, Fundación Instituto de Inmunología de Colombia (FIDIC), Carrera 50#26-20, Bogotá, Colombia; 2 School of Medicine and Health Sciences, Universidad del Rosario, Carrera 24#63C-69, Bogotá, Colombia; 3 Faculty of Natural and Mathematical Sciences, Universidad del Rosario, Carrera 24#63C-69, Bogotá, Colombia; 4 School of Medicine, Universidad Nacional de Colombia, Carrera 45#26-85, Bogotá, Colombia; 5 Mathematics Department, Universidad Pública de Navarra, 31006 Pamplona, Spain

**Keywords:** HR-HPV, Persistence, Clearance time, Colombia, Follow-up study, Viral load

## Abstract

**Background:**

The design of new healthcare schemes which involve using molecular HPV screening means that both persistence and clearance data regarding the most prevalent types of HR-HPV occurring in cities in Colombia must be ascertained.

**Methods:**

This study involved 219 HPV positive women in all of whom 6 types of HR-HPV had been molecularly identified and quantified; they were followed-up for 2 years. The Kaplan-Meier survival function was used for calculating the time taken for the clearance of each type of HPV. The role of a group of independent variables concerning the time taken until clearance was evaluated using a Cox proportional-hazards regression model or parametric (log-logistic) methods when necessary. Regarding viral load, the Wilcoxon rank-sum test was used for measuring the difference of medians for viral load for each type, according to the state of infection (cleared or persistent). The Kruskal-Wallis test was used for evaluating the change in the women’s colposcopy findings at the start of follow-up and at the end of it (whether due to clearance or the end of the follow-up period).

**Results:**

It was found that HPV-18 and HPV-31 types had the lowest probability of becoming cleared (1.76 and 2.75 per 100 patients/month rate, respectively). Women from Colombian cities other than Bogotá had a greater probability of being cleared if they had HPV-16 (HR 2.58: 1.51–4.4 95% CI) or HPV-58 (1.79 time ratio: 1.33-2.39 95% CI) infection. Regarding viral load, HPV-45-infected women having 1 × 10^6^ to 9.99 × 10^9^ viral copies had better clearance compared to those having greater viral loads (1.61 time ratio: 1.01-2.57 95% CI). Lower HPV-31 viral load values were associated with this type’s persistence and changes in colposcopy findings for HPV-16 gave the worst prognosis in women having low absolute load values.

**Conclusions:**

HPV infection clearance in this study was related to factors such as infection type, viral load and the characteristics of the cities from which the women came. Low viral load values would indicate viral persistence and a worse prognosis regarding a change in colposcopy findings.

## Background

Persistent infection with high-risk human papillomavirus (HR-HPV) types is the main (but not the only) cause of developing pre-cancerous lesions or cervical cancer [1-3].

Several types of HPV considered oncogenic have been found to be responsible for around 90% of cancer cases worldwide [3], having high HR-HPV type prevalence rates (i.e. HPV-16, −31, −18, −33, −45 and −58) amongst Colombian women [4]. Our group is aware that multiple infections are of great importance for our population, since more than 40% prevalence has been reported in some regions of the country [4].

It has been described that HPV infection represents a transient phenomenon throughout the whole world, leading to high infection prevalence, even though most cases do not produce cervical lesions and those which do, consist of low grade lesions involving spontaneous regression [5]. However, Colombia does have a high cervical cancer incidence rate and high morbidity-mortality values for a type of cancer which is highly preventable [6,7].

Intrinsic viral determinants, such as the infecting viral type or viral load, have been identified as HPV persistence markers [1,8,9]; however, some socio-environmental factors also play an important role in this type of infection and its possible outcomes [5,10].

The present work seeks to describe both viral and host factors which could be intervening in the persistence and clearance of the most common HR-HPV types in Colombia. This study was mainly aimed at providing epidemiological information illustrating how HPV infection can become eliminated in a target population, bearing the state of the infection (single or multiple) and the most prevalent HR-HPV types in mind.

## Methods

### Study population and ethical considerations

Women eligible for the present study were voluntarily attending cervical screening consultations in three Colombian cities (Chaparral, Girardot and Bogotá) between April 2007 and March 2010.

The present study involved 3 cities: Bogotá the country’s capital having an urban population, Chaparral located in the Tolima department is mainly inhabited by mestizos leading a sedentary life style and Girardot in the Cundinamarca department which has become a tourist destination due to its favourable weather and closeness to Bogotá. Data concerning Chaparral and Girardot was combined in a single category called “other city” to ensure a better analysis of women in this study, as both these cities are small, having similar climates and lying at less than 1,000 masl (Bogotá is 2,600 masl).

As inclusion criteria, all women signed a written informed consent form and completed a questionnaire regarding their sociodemographic characteristics, sexual behaviour and risk factor data before undergoing a gynaecological examination and providing a cervical smear sample. The signature of a parent or guardian was required for females younger than 18 years old. Women who stated that they did not intend to move from their home cities for at least 2 years after the study began were included in the follow-up study.

Amongst exclusion criteria considered, women who had negative HPV results, those whose samples had little DNA in them (to ensure that all PCR assays were performed satisfactorily) or no amplification for the *HMBS* gene were not included. Women who were pregnant at base line and who had less than 3 months or more than 9 months until their next visit were also excluded from the study.

The Papanicolaou test was used for analysing samples and HPV–DNA detection; real-time PCR was used for selecting just HPV positive women. This study was supervised and approved by the relevant ethics’ committees at Hospital de Engativá Nivel II (in Bogotá), Hospital San Juan Bautista (in Chaparral in the Tolima department) and Nuevo Hospital San Rafael (in Girardot in the Cundinamarca department).

### HPV DNA collection, processing and detection by PCR amplification

Cervical samples were collected with a cytobrush and kept in 95% ethanol at 4°C [11]. DNA from these samples was purified using a commercial Quick Extract Solution kit, following the manufacturer’s instructions. Samples were homogenised in 200 μL lysis buffer (10 mM Tris–HCl (pH 7.9), 0.45% Nonidet- P-40, 0.45% Tween 20 and 60 mg/mL proteinase K) and incubated for 6 min at 65°C, followed by 2 min at 92°C. Samples were centrifuged at 13,000 rpm for 10 min and the supernatant was removed and stored at 20°C.

Polymerase chain reaction (PCR) for human β-globin was carried out on samples to check DNA integrity using GH20/PC04 primers [12]. Three HPV generic primer sets were used for HPV-DNA detection, as described in previous studies (i.e. GP5+/GP6+, MY09/MY11 and pU1M/2R) [13].

### Viral load determined by real-time PCR

#### Primers and probes

Specific primers for each viral type and for *Homo sapiens* hydroxymethylbilane synthase (HMBS) were synthesised according to a study published by Moberg *et al*., [14]. The primers described by Moberg *et al.,* amplified the same region for HPV-33 and −58; a new set of primers aimed at the E7 region of these viral types was thus designed. Designing probes for each viral type and HMBS was based on four parallel duplex real-time PCRs per patient, taking into account the types included in each reaction (Table 1) and support by integrated DNA technologies.

**Table 1:** Primers and probes used for qPCR

**Region**	**Viral type**	**Primer sequence 5′ - 3′**	mT*	**Plasmid product quantification**	**qPCR test**	**Probe**	**Probe size (bp)**	**Quencher**
E7	HPV-16	AGCTCAGAGGAGGAGGAT	54	1.43E10^12^	Reaction 1	FAM	78	ZEN/Iowa Black FQ
		GGTTACAATATTGTAATGGGCTC						
E1	HPV-18	CATTTTGTGAACAGGCAGAGC	53.7	1.19E10^12^	Reaction 2	Cy5	80	IBRQ
		ACTTGTGCATCATTGTGGACC						
E6	HPV-31	ACGATTCCACAACATAGGAGGA	53.7	1.35E10^12^		HEX	78	ZEN/lowa Black FQ
		TACACTTGGGTTTCAGTACGAGGT						
E7	HPV-33	ATTAAGTGACAGCTCAGATGA	53.7	1.86E10^12^	Reaction 3	FAM	78	ZEN/Iowa Black FQ
		ACATAAACGAACTGTGGTGTT						
E1	HPV-45	CCATTTGTGAACAGGCAGAGC	53.7	1.59E10^12^		Cy5	76	IBRQ
		CAACACCTGTGCATCATTCTGA						
E7	HPV-58	CGAGGATGAAATAGGCTTGG	53.7	1.23E10^12^	Reaction 4	HEX	109	ZEN/Iowa Black FQ
		ACACAAACGAACCGTGGCGT						
HMBS		GCCTGCAGTTTGAAATCAGTG	53.7	1.98E10^12^		FAM	76	ZEN/Iowa Black FQ
		CGGGACGGGCTTTAGCTA						

*HPV* Human papillomavirus, *mT* Melting temperature in °C, *FAM* 6-carboxyfluorescein, *Cy5* FluoroLink Mono Reactive Dye Cy5, *HEX* Hexachlorofluoresceine, *HMBS*, Hydroxymethylbilane synthase.

*Melting temperature for quantitative real-time PCR.

#### Cloning and sequencing

Processed samples were used as template for PCR (10 μL final volume), containing 0.5 U/μL Mango *Taq* DNA polymerase (Bioline), 1× Mango *Taq* Color reaction Buffer, 2 mM MgCl_2_, 250 nM dNTPs, 1 mM of each primer and DNase-free water to fulfil the necessary reaction volume. The PCR protocol for each fragment consisted of initial denaturing for 5 min at 95°C, followed by 35 cycles of 30 s at 95°C, 20 s at corresponding melting temperature and 30 s at 72°C. A reaction containing DNA-free water was used as negative control. The amplicons so obtained were purified with a Wizard PCR preps kit (Promega), once their quality has been evaluated on 3.25% agarose gel. A TOPO TA cloning kit was used for ligation, followed by transformation in TOP10 *E. coli* cells (Invitrogen). Several clones which grew on selective LB plates with 50 μg/mL kanamycin were incubated in LB broth at 37°C with 250 rpm overnight. Recombinant plasmids were purified using an UltraClean mini plasmid prep kit (MO BIO laboratories, California, USA) and sequenced with an automatic ABI PRISM 310 Genetic Analyzer (PE Applied Biosystems, California, USA). Each insert’s integrity was checked by aligning the products with the respective theoretical sequenced fragments of each gene using Clustal W software [15].

#### Real-time PCR

A NanoDrop 2000 (Thermo Scientific NanoDrop Products) was used for quantifying plasmid DNA and the DNA copy numbers were calculated by using the URI Genomics and Sequencing Center web site [16] (Table 1). Standardised RT-PCR assays with 10-fold serial plasmid dilutions (10^11^-10^6^copies) gave a standard curve for each viral type and HMBS gene (−3.2 to −3.5 slope values). Samples were tested for HPV-16, HPV-18, HPV-31, HPV-33, HPV-45 and HPV-58. The human HMBS (hydroxymethylbilane synthase) gene was amplified in all samples to verify DNA integrity and calculate viral copy number per cell. PCR involved using a CFX96 Touch Real-Time PCR detection system which can detect 6 different fluorescent dyes; four real-time PCR reactions were carried out per sample, one for detecting HPV-16, a second for HPV-18 and −31, a third for HPV-33 and −45 and a fourth one for HPV-58 and HMBS.

The HPV-16 PCR mix contained 1× reaction buffer, 1.5 mM MgCl_2_, 250 nM of each dNTP, 250 nM of each primer, 1.5 U/μL MangoTaq Polymerase (Bioline) and 500 nM probe. The second PCR mix for HPV-18 and −31 consisted of 1× reaction buffer, 2 mM MgCl_2_, 275 nM of each dNTP, 500 nM HPV-18 primers, 250 nM HPV 31 primers, 1.5 U/μL MangoTaq polymerase and 500 nM of each probe. The HPV-33 and −45 PCR mix included 1× reaction buffer, 1.75 mM MgCl_2_, 275 nM of each dNTP, 250 nM HPV-33 primers, 500 nM HPV-45 primers, 1.5 U/μL MangoTaq polymerase and 500 nM of each probe. The last PCR mix for HMBS and HPV58 contained 1× reaction buffer, 1.87 mM MgCl_2_, 250 nM of each dNTP, 500 nM of each primer, 1.5 U/μL MangoTaq polymerase, 200 nM HMBS probe and 500 nM HPV 58 probe. All real-time PCR reactions contained 2 μL of the DNA extracted from each cervical sample and DNA-free water to complete 20 μL volume.

96-well plates were used for each run, including 6 standards for each viral type and HMBS, involving 10-fold plasmid dilutions (10^11^-10^6^ copy dynamic detection range) and a no template control to rule out DNA contamination.

The thermal cycling conditions for HPV-18, −31, −33, −45, −58 and HMBS consisted of initial denaturing for 5 min at 94°C, followed by 30 amplification cycles for 10 s at 94°C and 30 s at 53.7°C. Initial HPV-16 denaturing was followed by 30 PCR cycles for 30 s at 54°C and 30 s at 94°C.

Viral load values were given as absolute and normalised. The viral load was normalised to cellular DNA input amount, using the following formula: viral load (HPV copies/cell): number of HPV copies/(number of HMBS copies/2) [17].

### Statistical analysis

Women who had had both a Pap-smear result and HPV-DNA detected by PCR and who fulfilled the follow-up inclusion criteria (at least 3 follow-ups, leaving 6 to 9 months between visits) were included in the analysis. Women were excluded where the HMBS gene was not amplified by RT-PCR. Analysis was based on type-specific HPV infection rather than on individual women, taking into account that multiple infection is common in the Colombian population [4].

Cox’s multivariate regression model was used when calculating sample size; hazard ratios (HR) of at least 2 were thus considered, whenever they had a 5% significance level, 80% power, 0.55 standard deviation of tested covariates and 0.1 correlations between tested covariates. The probability of clearance was set at 0.7, according to previous reports [18,19]. Such suppositions required sample size of at least 86 women. STATA 12 stpower command was used for making the calculations.

Clearance was defined as at least two consecutive type-specific HPV DNA samples proving negative, such samples taken at 6-month intervals following a positive sample [20]. Persistence was defined as the identification of the same HPV type in baseline and follow-up samples [21]. The time taken for HR-HPV infection clearance was calculated in months (95% CI), estimated using the Kaplan-Meier survival function.

The first step was evaluating each variable independently to assess their importance regarding clearance time (Table 2); those variables having a significance level of less than 0.2 in the univariate analysis were included in the multivariable models.

**Table 2:** Baseline characteristics’ distribution according to HPV infection stage

**Baseline characteristics**		**Single HPV infection n = (%)**	**Multiple HPV infection n = (%)**	**Total**	**p =**
**Age (y) (n = 219)**	<30	10 (31.25 )	22 (68.75)	32	0.54
	30-50	31 (22.96)	104 (77.04)	135	
	>50	11 (21.15 )	41 (78.85)	52	
**Origin (n = 219)**	Bogota	21 (30.43)	48 (69.57)	69	0.115
	Other cities	31 (20.67)	119 (79.33)	150	
**No. of family members living together (n = 219)**	<=4	35 (24.65 )	107 (75.35)	142	0.670
	>4	17 (22.08 )	60 (77.92)	77	
*Average monthly income (n = 219)	Minimum	30 (20.98)	113 (79.02)	143	0.243
	>minimum	22 (28.95)	54 (71.05)	76	
**Ethnicity (n = 219)**	Indigenous	0	1 (100)	1	1
	Mestizo	52 (24.19)	163 (75.81)	215	
	Afro-descendant	0	3 (100)	3	
**Marital status (n = 219)**	Married	2 (14.29)	12 (85.71)	14	0.849
	Divorced	1 (33.33)	2 (66.67)	3	
	Single	1 (20)	4 (80)	5	
	Living with partner	48 (24.49)	148 (75.51)	196	
	Widow	0	1 (100)	1	
**Healthcare scheme affiliation (n = 219)**	Contributory	6 (35.3)	11 (64.70)	17	0.244
	Subsidised	46 (22.77)	156 (77.23)	202	
**Age on first intercourse (n = 219)**	<18	22 (23.66)	71 (76.34)	93	0.979
	≥18	30 (23.81)	96 (76.19)	126	
**Lifetime sexual partners (n = 219)**	1	25 (24.51)	77 (75.49)	102	0.804
	>1	27 (23.08)	90 (76.92)	117	
**Contraceptive method (n = 219)**	No method used	25 (27.78)	65 (72.22)	90	0.470
	Surgery	14 (21.21)	52 (78.79)	66	
	Hormonal	3 (13.04)	20 (86.96)	23	
	Barrier	10 (25)	30 (75)	40	
**Number of pregnancies (n = 219)**	None	2 (50)	2 (50)	4	0.07
	1-2	25 (28.09)	64 (71.91)	89	
	3-4	15 (16.13)	78 (83.87)	93	
	>4	10 (30.3)	23 (69.7)	33	
**Abortions (n = 158)**	None	23 (28.05)	59 (71.95)	82	0.481
	1	11 (20)	44(80)	55	
	> = 2	4 (19.05)	17 (80.95)	21	
**STD (n = 210)**	No	37 (22.16)	130 (77.84)	167	0.112
	Yes	15 (34.88)	28 (65.12)	43	
**Colposcopy results (n = 202)**	L-SIL	14 (29.17)	34 (70.83)	48	0.819
	H-SIL	0	1 (100)	1	
	Negative	37 (24.18)	116 (75.82)	153	
**Cytological findings (n = 219)**	ASC-US	1 (11.11)	8 (88.89)	9	0.840
		L-SIL	3 (25)	9 (75)	12
		Negative	48 (24.24)	150 (75.76)	198

*HPV* Human papillomavirus, *p=* P value, *STD* Sexually-transmitted diseases, *ASCUS* Atypical squamous cells of undetermined significance, *SIL* Squamous intraepithelial lesions. Some variables had lower values due to loss of data regarding self-completed questionnaires.

*The minimum average monthly income in Colombia is about US$ 300.

The independent variables included in the multivariable model were city, ethnicity, age of first sexual relationship, number of lifetime sexual partners, family planning method, coinfection and viral load (categorised as low, viral load being lower than 9.99E + 5, middle viral load between 1.00E + 6 to 9.99E + 9 and high viral load being higher than 1.00E + 10) concerning time taken to clearance using a Cox’s proportional hazard (PH) regression model or parametric methods (log-logistic) when the PH assumption was violated. The PH assumption was graphically evaluated using log-log plots and a PH test based on weighted residuals using Grambsch and Therneau tests [22]. The choice of parametric model was defined using Akaike information criterion (AIC) and Bayesian information criterion (BIC).

Three categories were assigned to the variable “changes in colposcopy” (alike, improved and worsened) for evaluating changes in colposcopy findings between the results of colposcopy at the start of follow-up and the end of it (whether due to clearance of the virus or not). The Kruskall-Wallis test was used for evaluating the difference in viral load for each viral type and change in colposcopy findings since the sample did not have a normal distribution. The Mann–Whitney test was also used for evaluating viral load according to the state of infection (persistent or cleared).

Categorical variable distribution amongst groups was assessed by Chi-squared test or Fisher’s test, as appropriate. Median and interquartile ranges were used for quantitative variables, according to the data distribution. Incidence ratios were estimated using months of follow-up as denominator. A ≤ 0.05 p value was considered statistically significant; STATA 12 was used for all statistical analysis.

## Results

The present work has consolidated data concerning 219 women infected by several HR-HPV types; they became voluntarily incorporated into our follow-up study. All the women included guaranteed to attend a base-line visit and at least 3 follow-up visits with around 6 month difference (±3 months); 23.3% of the population being sampled managed to attend follow-up 4 (i.e. data became available from 5 visits).

Regarding baseline information, 23.7% (n = 52: 18.3-29.9 95% CI) of the women in the study were infected by a single type of HR-HPV (single infection); the rest of the population, 76.3% (n = 167) had multiple infections, distributed as follows: 26% (n = 57: 20.3-32.4 95% CI) had infections having simultaneous detection for 2 types of HR-HPV, 29.7% (n = 65: 23.7-36.2 95% CI) infection by 3 types of HR-HPV, 13.2% (n = 29: 9.1-18.4 95% CI) had positive identification for 4 viral types, 5% (n = 11: 2.5-8.8 95% CI) were infected by 5 high risk types and 2.3% (n = 5: 1–5.2 95% CI) had viral DNA identification for 6 types of HR-HPV.

Participants’ age ranged from 17 to 71 years-old (SD 10.8, mean 42.2 years). Most of the participating population was mestizo (98.2%; n = 215: 95.4-99.5 95% CI), and from the city of Girardot (66.2%; n = 145: 59.6-72.4 95% CI). The population’s socio-demographic and sexual behaviour data was described regarding follow-up (base-line), according to the state of the infection (single infection or multiple infections; Table 2).

**Figure 1:**
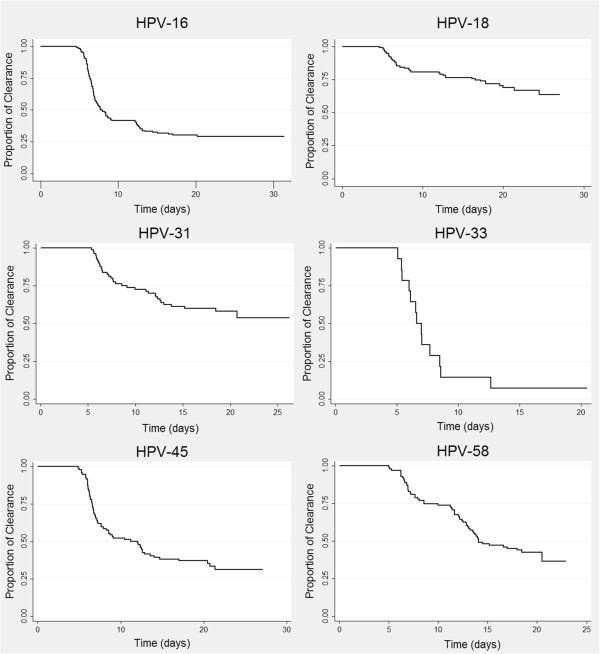
Kaplan-Meier plots of time taken to HPV infection clearance for the 6 HR-HPV types studied here.

**Figure 2:**
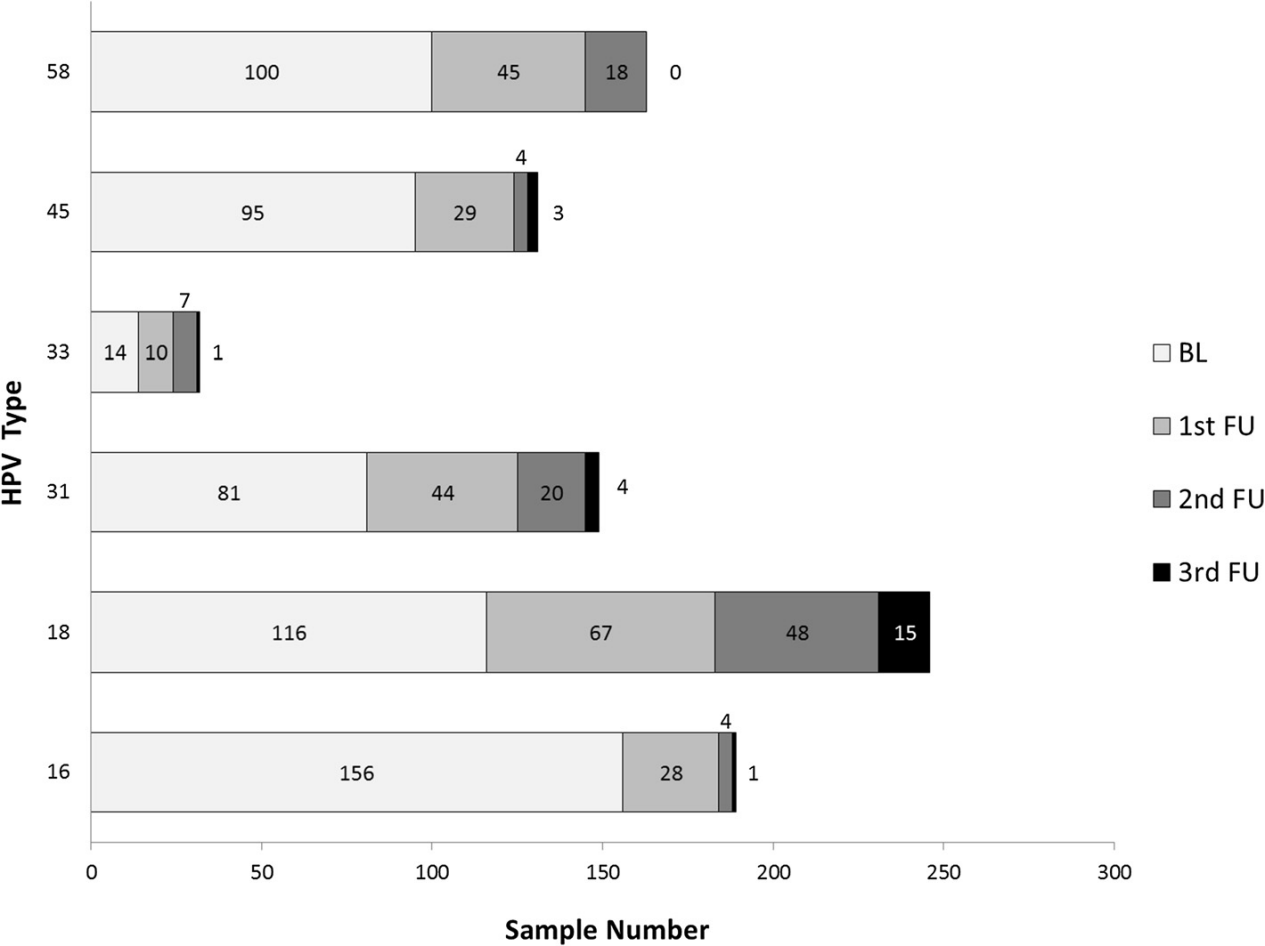
**Persistence rates for the 6 HR-HPV types.** BL: baseline; 1st FU: first follow-up; 2nd FU: second follow-up; 3rd FU: third follow-up.

Survival data was estimated for each type of HR-HPV regardless of single infection or multiple infections and clearance time for each type (Figure 1). A greater clearance occurred for HPV-33-infected women, followed by HPV-16-infected females, whilst fewer events per month occurred for HPV-18- and HPV-31-infected women (Table 3).Figure 2 clearly shows that HPV-18 infection was the most persistent; infection had not become resolved in 15 women 2 years later, followed by HPV-31 which was present in 4 women having positive identification for this type during each follow-up visit (infection remaining unresolved by the end of the study).

**Table 3:** Prevalence, reinfection and clearance rates concerning the 6 HR-HPV types

**Viral type**	**Baseline**			**Clearance**			**Reinfection**		
	**Prevalence% (n=) [95% CI]**	**Viral load, median (IQR)**		Rate* [95% CI]	Total HPV viral load**, median (IQR)		**% (n=) [95% CI]**	**Viral load, median (IQR)**	
		**Absolut**	**Normalised**		**Absolut**	**Normalised**		**Absolut**	**Normalised**
**HPV-16**	71.2% (156) [64.7-77.1]	2.9E + 6 (9.7E + 6)	0.2 (6.7)	5.9 [4.9-7.2]	2.17E + 09 (7.67E + 10)	5 (13,468)	48.6% (53) [39–58]	2.9E + 6 (9.2E + 6)	0.32 (5.3)
**HPV-18**	53% (116) [46.1-59.7]	4.3E + 6 (4.56E + 07)	0.42 (13)	1.76 [1.3-2.4]	6.56E + 09 (6.34E + 11)	208 (30,101)	12% (7.4-20.4)	3.0E + 6 (4.28E + 07)	0 .1 (1.8)
**HPV-31**	37% (81) [30.6-43.8]	9.04E + 07 (6.29E + 09)	44 (3,360)	2.75 [1.96-3.84]	1.23E + 07 (3.02E + 07)	7 (96)	50% (17) [32–67]	1.96E + 09 (7.61E + 09)	3,360 (12,400)
**HPV-33**	6.4% (14) [3.5-10.5]	2.96E + 07 (1.14E + 09)	3.6 (39,100)	11.51 [6.7-19.8]	1.39E + 9 (1.91E + 10)	774 (4,683)	38.5% (5) [14–68]	1.47E + 08 (5.82E + 08)	51 (128)
**HPV-45**	43.4% (95) [36.7-50.2]	2.48E + 06 (1.12E + 09)	0.33 (180)	5 [3.9-6.42]	1.84E + 08 (9.30E + 11)	18 (1E + 05)	43.5% (27) [30.9-56.7]	1.14E + 08 (1.94E + 09)	17 ( 7,180)
**HPV-58**	45.7% (100) [38.9-52.5]	6.14E + 05 (2.1E + 06)	0.4 (15.7)	4.05 [3.1-5.24]	4.35e + 08 (5.92e + 10 )	141 (5,749)	0	0***	0

*Clearance rates are given in values per month, per 100 individuals.

**Total HPV viral load was taken as the mean of the other HPV type infections on clearance.

***The load for other HPV-58 types was not calculated as there was no re-infection.

*HPV* Human papillomavirus, *IQR* Interquartile range.

Specific viral load type values were also determined in this study; Table 3 gives both absolute and normalised viral load values for each type infections at the start of the study. It is worth highlighting that those infected by HPV-31 had the highest viral load values, even those normalised by the number of cells, whilst HPV-16 gave the lowest viral load values.

Hazard ratios were calculated for two (HPV-16 and-18) of the 4 most prevalent viral types in molecular determination (i.e. HPV-16, −18, −45, −58), bearing in mind the most important variables in univariate models, for each type (data not shown) (Table 4). Time ratios were calculated for the remaining types, since these (HPV-45 and −58) did not comply with supposed proportional risks for the aforementioned variables; variables could not thus be re-categorised nor could they be assumed to be time-dependent variables. Regression was thereby modelled using a parametric model, bearing in mind the shape of the hazard function and AIC and BIC. The log-logistic model gave the best fit for both types with the foregoing criteria.

**Table 4:** Determinants of clearance for the most prevalent HR-HPV types

**Time independent variables**		**HPV-16**	**HPV-18**	**HPV-45**	**HPV-58**
		**Multivariable model HR (95% CI)**	**Multivariable model HR (95% CI)**	**Multivariable model Tm R (95% CI)**	**Multivariable model Tm R (95% CI)**
**Age**	**>50**	0.99 (0.53-1.86)	0.59 (0.18-1.87)	1.13 (0.63-2.02)	0.94 (0.63-1.38)
	**35-50**	1.14 (0.66-1.96)	0.79 (0.35-1.75)	0.77 (0.49-1.20)	1.26 (0.89-1.79)
	**<35**	*Reference*	*Reference*	*Reference*	*Reference*
**City**	**Other city**	**2.58 (1.51-4.4)**	1.21 (0.51-2.9)	0.97 (0.62-1.52)	**1.79 (1.33-2.39)**
	**Bogota**	Reference	Reference	Reference	Reference
**Ethnicity**	**Afrodescendant**	2.35 (0.69-7.93)	0.85 (0.11-6.61)	0.42 (0.16-1.12)	-
	**Mestizo**	*Reference*	*Reference*	*Reference*	*Reference*
	Other*	-	-	-	1.17 (0.47-2.89)
**Contraceptive method**	**No method**	1.01 (0.65-1.59)	0.75 (0.33-1.73)	0.84 (0.56-1.27)	0.98 (0.73-1.30)
	**Hormonal**	1.45 (0.72-2.96)	1 (0.35 -2.9)	0.76 (0.45-1.26)	0.97 (0.54-1.72)
	**Other**	*Reference*	*Reference*	*Reference*	*Reference*
**Lifetime sexual partners**	**1**	1.24 (0.83-1.86)	1.2 (0.61-2.51)	1.05 (0.72-1.52)	1.10 (0.84-1.44)
	**>1**	*Reference*	*Reference*	*Reference*	*Reference*
**Age at first Intercourse**	**≥18**	0.93 (0.6-1.41)	1.08 (0.53-2.19)	1.23 (0.85-1.77)	1.13 (0.85-1.51)
	**<18**	*Reference*	*Reference*	*Reference*	*Reference*
**Coinfection**	**Yes**	0.87 (0.46-1.65)	2.49 (0.54-11.45)	1.06 (0.46-2.45)	0.87 (0.58-1.32)
	**No**	*Reference*	*Reference*	*Reference*	*Reference*
Viral Load**	**Low**	2.1 (0.87-5.15)	0.48 (0.08-2.93)	1.27 (0.76-2.12)	1.36 (0.81-2.29)
	**Middle**	1.49 (0.64-3.51)	0.74 (0.24-2.27)	**1.61 (1.01-2.57)**	1.04 (0.61-1.78)
	**High**	*Reference*	*Reference*	*Reference*	*Reference*

*Since only 2 women of other ethnicity had HPV-58 (1 Afrodescendant and 1 Indigenous) they were put in a category “Other”, just for this type.

**Low viral load meant a viral load below 9.99E + 5, middle 1.00E + 6 to 9.99E + 9 and high meant a viral load higher than 1.00E + 10.

Values in bold = p < 0.05.

*HPV* Human papillomavirus, *CI* Confidence interval, *HR* Hazard ratio, *Tm R* Time ratio.

Multivariate model values showed that the probability of clearance when a woman had HPV-16 or HPV-58 infection became significantly increased in women from another city compared to women living in the capital. It is worth stating that both types belong to the same species (A9).

Regarding the types belonging to species A7, HPV-18 infection did not have a statistically significant association with any variable evaluated here. However, it was observed that the probability of clearance regarding HPV-45 became significantly increased when the absolute viral load for this type ranged from 1 × 10^6^ to 9.99 × 10^9^ compared to loads equal to or greater than 1 × 10^10^.

It should be highlighted that single or multiple HR-HPV infection was not associated with time to clearance in the present sample, since coinfection values for any HPV type were not statistically significant in this model (Table 4).

When evaluating the medians for normalised viral load (per cell) for each type, according to the state of infection at the end of follow-up (cleared or persistent), it was observed that the median for HPV-31 type in the group of women where clearance was found (median = 332.5; IQR = 12,399.72) was greater than the median for those where this virus was not cleared (median = 9.4; IQR = 1,659.98, p = 0.0450). There were no differences in any of the groups regarding the medians for the other HR-HPV types.

**Figure 3:**
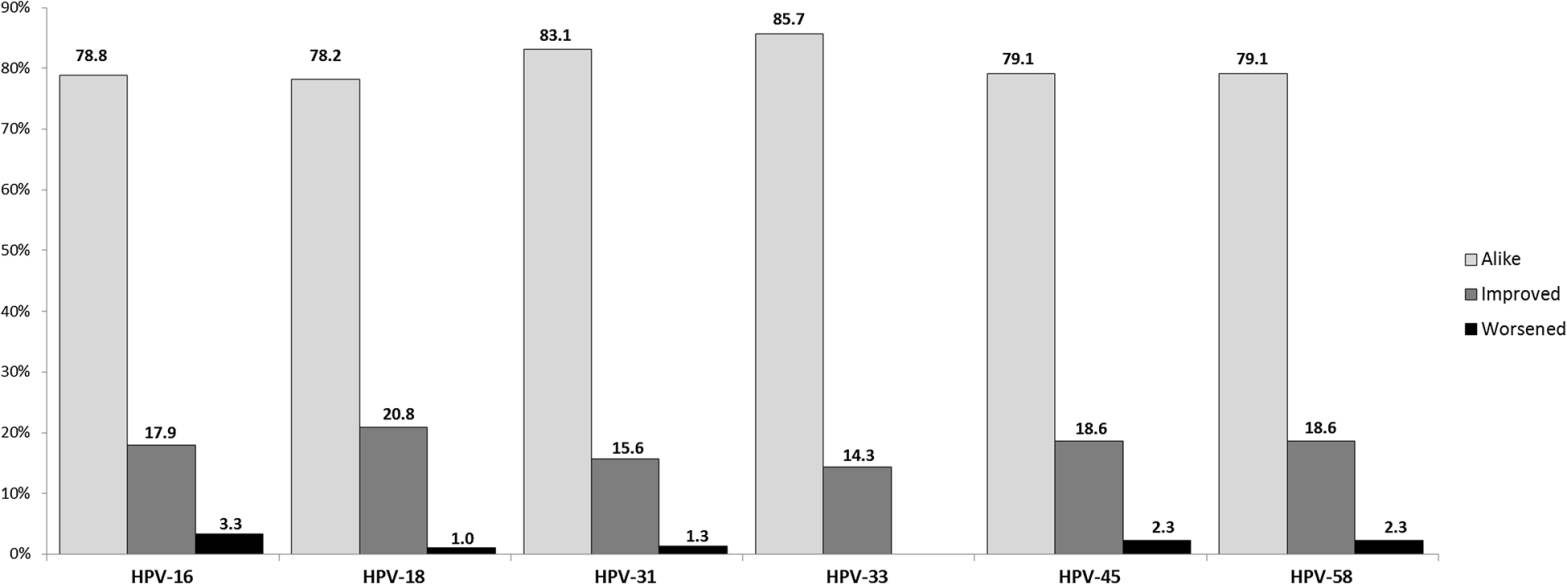
Change in colposcopy results for the 6 HR-HPV types studied here.

The change in colposcopy findings was also evaluated concerning the result at the start of follow-up and the result at the moment of clearance, or at the end of follow-up (Figure 3). There were statistically significant differences regarding HPV-16 concerning absolute values for viral loads for each group, since the value for the group which became worse regarding diagnosis by colposcopy was lower (median = 89,300; IQR = 253,600) than that for the other groups (improved: median = 2.9 × 10^6^; IQR = 1.1 × 10^7^; alike: median = 2.9 × 10^6^; IQR = 9.9 × 10^6^) (p = 0.046). There were no differences in the rest of the types evaluated here regarding viral load according to change in colposcopy findings.

When evaluating normalised viral load according to the degree of HPV infection and colposcopy findings at the start and end of follow-up, it was found that median viral load for HPV-58 was greater for women who had a better prognosis (median = 3.98; IQR = 351.9) compared to those whose prognosis remained the same (alike) (mean = 0.013; IQR = 0 .68), only in the group of women having persistence for this virus (p = 0.012).

The number of reinfections for each viral type was determined (Table 3); HPV-58 was the only viral type for which there were no reinfection events during the time follow-up lasted.

## Discussion

This study has provided detailed epidemiological data for six HR-HPV types present in a Colombian cohort. This has been the first study in Colombia (to the best of our knowledge) aimed at using real time quantification of DNA from the 6 most prevalent types of HR-HPV, giving absolute and normalised load values.

Findings concerning the virus’ persistence for the 6 types included here have demonstrated that the risk of acquiring a later HPV infection becomes increased in women already infected by any type of HPV (regardless of complying with a phylogenetic relationship [23], mainly between high risk types [24,25]). Such coinfection could have been the result of immune system deficiency regarding clearance, thereby facilitating viral persistence at the infection site [25].

Regarding the state of infection (single or multiple) and its relationship to clearance time, this work did not reveal an important relationship between such aspects. Various studies have shown that type specific HPV clearance seems to occur regardless of coinfection in an immunocompetent population [26,27].

The highest clearance rates in our cohort were observed for HPV-33 and HPV-16. Previous reports have shown that HPV-16 clears out after other HR-HPV types [26,28,29], but in or study, this type of infection displayed a more transient pattern. Our results are in agreement with previous studies showing that the majority of women with a type-specific infection are negative for that particular viral after one year [19,30].

The reduced clearance rates observed for infections with HPV-18 and −31 types is particularly important bearing in mind that HPV-31 was found in high prevalence in our country [4] and that HPV-18 has been detected in aggressive forms of cancer [31].

Despite HPV-31 was not evaluated in the multivariable model due to the low sample size, it is worth noting that it displayed the highest viral load values and one of the lowest clearance rates. Future studies analysing more women infected with this viral type might help to a better understanding about the influence of viral loads in the type-specific clearance process.

HPV-16, −18 and −58 viral load values did not have a clear relationship with clearance time. This has already been shown for a population from Bogotá in a study involving semi-quantitative identification of viral DNA [24]. The present study showed that, regardless of using a more sensitive technique, no relationship was established between viral load and clearance time for these types, not just in Bogotá, but also in other Colombian cities.

Another factor associated with clearance time was city of origin, showing that women infected by types from the A9 species became cleared more rapidly if they came from Girardot/Chaparral. It is supposed that these cities have factors related to sexual behaviour or cultural characteristics which were not measured in this study and which would have modulated such findings. When analysing the control arm of the large randomised PATRICIA study, it was found that region of origin was one of the behavioural determinants of clearance time, as north-American women took less time to clearance than their European counterparts [20].

Regarding ethnicity, fine control was not used for obtaining it; thus, other ethnic characteristics which were not controlled in this study may have intervened in such marked association between city and clearance time. Another aspect concerned the women’s nutritional state; most women were from low socio-economic strata. However, nutritional and/or feeding data was not controlled and may have provided more detailed characteristics concerning the population’s idiosyncrasies. Previous studies have shown that women who consumed one or more servings of vegetables per day cleared their HPV infections more quickly than women who did not consume vegetables daily [32]. The intake of lower levels of micronutrients found in vegetables has been associated with increased persistence [33].

Other factor that was not measured in this study but that could influence HPV clearance is hygienic practices. In a cohort of university students, it has been shown that the use of tampons was associated with a reduced rate of HR-HPV clearance [32].

Very interesting data for three A9 species types (HPV-16, −31 and −58) was revealed when determining viral load according to the state of infection and colposcopy findings since these had low viral load values (absolute or per cell) associated with greater lesion severity at the end of follow-up or when infection did not become eliminated. Besides intermediate viral load values for HPV-45 (A7 species) were associated with faster time to clearance, this may be a factor related to transient infection. Such results could have been due to immune system evasion mechanisms since it has been reported that low HPV viral load values have been related to persistent infection [34] and it could be suggested that higher viral load values could be detected efficiently by the immune system and rapidly eliminated.

This is contradictory with studies proposing that high viral loads facilitate persistence, specifically, it has been shown that HPV-16 viral loads in LSIL and HSIL were higher compared with no intraepithelial lesion or malignancy [35].

This work has several strengths, such as having compiled data from two important focuses of HPV infections (i.e. Girardot and Bogotá), determined viral load using the most sensitive technique for doing so and the percentage of multiple infections revealing an important Colombian populational characteristic. However, the study had difficulties in terms of follow-up times, since infection transience meant that shorter follow-up times than the ones established here may probably have led to obtaining more precise clearance and incidence values. Our information was limited to using prevalent high risk infections for analysing persistence and clearance of infection; the foregoing means that follow-up studies are needed to facilitate understanding the most prevalent epidemiological HPV patterns for Colombia.

Diagnosing HPV infection in clinical specimens has been widely accepted to date in Colombia; viral DNA identification in this type of sample has been included in the Obligatory Healthcare Plan, 2012. The following step must thus be to incorporate monitoring from the identification of HPV infection in cervical cancer control schemes. Such work thus contributes towards the search for a correct algorithm for defining HPV DNA screening since the time taken for most women to clear the virus must be determined for calculating the determinants of such scheme and be referred to regular monitoring [19].

## Conclusions

Time to clearance in Colombian females infected by the most frequently occurring HR-HPV types in the sample population was not modulated by infection status (single or multiple). However, viral load played a role in terms of infection regarding HPV-45 and the origin of HPV-16 and −58 infection. Viral persistence and worsening of cytological findings were related to lower HPV-16, −31 and −58 viral loads. All women in our sample who eliminated HPV-58 were not infected again by this viral type. Given that time to clearance was related to lesion development, such information should prove significant when designing HPV DNA primary screening in Colombian healthcare systems, as well as in developing countries.

## Abbreviations

HPV: Human papillomavirus; HR-HPV: High risk human papillomavirus; L-SIL: Low squamous intraepithelial lesion; H-SIL: High squamous intraepithelial lesion; ASCUS: Atypical squamous cells of undetermined significance; HMBS: Hydroxymethylbilane synthase; PCR: Polymerase chain reaction; RT-PCR: Real-time PCR; DNA: Deoxyribonucleic acid; STD: Sexually-transmitted diseases; SD: Standard deviation; CI: Confidence interval; HR: Hazard ratio; Tm R: Time ratio; IQR: Interquartile range; AIC: Akaike information criterion; BIC: Bayesian information criterion.

## Competing interests

The authors declare that they have no competing interests.

## Authors’ contributions

All authors contributed ideas to this paper and reviewed the manuscript for important intellectual content. SCSDL and MC provided the concept, acquired, analysed and interpreted data, designed the study and wrote the manuscript. LDRO and DAMP developed the methodology and were involved in drafting the manuscript. RS provided statistical analysis, interpreted data and helped in writing the manuscript. The study was supervised by APP, MEP and MAP who provided expertise regarding the discussion of results. All authors read and approved the final manuscript.

## Pre-publication history

The pre-publication history for this paper can be accessed here:

http://www.biomedcentral.com/1471-2334/14/395/prepub
